# Macular Vessel Density Changes in Young Adults With High Myopia: A Longitudinal Study

**DOI:** 10.3389/fmed.2021.648644

**Published:** 2021-06-08

**Authors:** Ya Shi, Luyao Ye, Qiuying Chen, Guangyi Hu, Yao Yin, Ying Fan, Jianfeng Zhu, Jiangnan He, Zhi Zheng, Haidong Zou, Xun Xu

**Affiliations:** ^1^Department of Ophthalmology, Shanghai General Hospital, Shanghai Jiao Tong University, National Clinical Research Center for Eye Diseases, Shanghai Key Laboratory of Ocular Fundus Diseases, Shanghai Engineering Center for Visual Science and Photo Medicine, Shanghai Engineering Center for Precise Diagnosis and Treatment of Eye Diseases, Shanghai, China; ^2^Department of Preventative Ophthalmology, Shanghai Eye Disease Prevention and Treatment Center, Shanghai Eye Hospital, Shanghai, China

**Keywords:** macular vessel density, high myopia, optical coherence tomography angiography, retinal thickness, longitudinal study

## Abstract

**Background:** To characterize the longitudinal changes of macular vessel density in young adults and its associated factors.

**Methods:** The right eyes of 309 participants (75 high myopic, 194 mild-to-moderate myopic, and 40 healthy) were followed up for 21 months. OCTA images were acquired at two visits using follow-up scans. Macular vessel density was calculated globally and in the nine early treatment diabetic retinopathy study (ETDRS) subfields of the macula superficial layer.

**Results:** The macular vessel density significantly decreased in young myopes after a 21-month follow up (*p* < 0.05), with variations among sectors. Compared with healthy eyes, HM group exhibited a faster reduction in global macular vessel density (*p* = 0.0307) as well as in sectors of inner-inferior (II), inner-temporal (IT), and outer-temporal (OT) (all *p* < 0.05). Multivariate regression analysis showed that longer baseline axial length (AL) was significantly associated with larger reduction of macular vessel density in the inner-inferior, inner-temporal and outer-temporal sectors (all *p* < 0.05).

**Conclusions:** Compared with emmetropes, high myopes presented greater loss of macular vessel density over time in global and in the inner-inferior, inner-temporal and outer-temporal sectors. A longer baseline AL was associated with larger changes of macular vessel density in the inner-inferior, inner-temporal and outer-temporal sectors.

## Introduction

Myopia has become a serious public health concern due to its significantly increasing prevalence, especially in East Asia (1–4). As a leading cause of vision loss worldwide (5), high myopia (HM) may result in retinal disorders, including lacquer crack formation, Forster-Fuchs' spots, chorioretinal atrophy, choroidal neovascularization, foveoschisis, and posterior staphyloma (6, 7). These complications are highly associated with morphological changes of retinal vessels (8, 9). Therefore, the change of retinal microvasculature in HM has been an important issue for several decades, which may provide a critical clue for understanding the pathophysiology of HM-associated diseases. It was previously reported that in the patients with HM, reduced retinal vessel density (10) and blood flow (11) were evident in the large retinal vessels visible on fundus photographs. Moreover, decreased choroidal blood flow not only has been found to be associated with increase of axial length (AL), but also may be a possible indication for progressive myopia (12). Recently, in retinal microvasculature, studies pointed out that students with HM experienced decreased deep perifoveal vessel density and radial peripapillary capillary (13, 14). Hence, investigating the longitudinal changes in macular microvasculature and their interaction in young adults with high myopia prior to retinal damage may reveal the underlying pathophysiology of the disorder and assist to implement an effective treatment or prevention.

Optical coherence tomography angiography (OCTA), a newly developed imaging modality, has assisted scholars to quantitatively and qualitatively measure retinal and choroidal microvasculature non-invasively (15). The present study aimed to assess changes of macular vessel density using OCTA in young adults with HM, and investigate the associations between the changes of vessel density and ocular parameters through a longitudinal study.

## Methods

### Study Participants

The participants in this longitudinal study, who were randomly selected from students of the Shanghai University, had been examined in October 2016 and followed up in July 2018. The study methodology of baseline examination has been previously described in detail (13). In brief, 760 participants aged 16–28 years with spherical equivalent (SE) <0.5 D, best-corrected visual acuity (BCVA) ≥ 20/25, intraocular pressure (IOP) ≤ 21 mmHg, normal anterior chamber angles, normal optic nerve head without glaucomatous changes, and no retinal nerve fiber layer abnormalities, were enrolled. The exclusion criteria were eyes with other ocular diseases (congenital cataract, glaucoma, and retinopathy), previous intraocular or refractive surgery, and any systemic diseases (hypertension and diabetes). This study was approved by the Ethics Committee of Shanghai General Hospital, Shanghai Jiao Tong University (Shanghai, China), and was conducted in accordance with the tenets of the Declaration of Helsinki. Informed consent forms were obtained from all the study subjects. The study protocol was registered at Clinical Trials.gov PRS (Registration No. NCT03446300).

### Study Procedures

All participants attended two visits and underwent comprehensive eye examinations at each time, including refractive error assessment using an autorefractor machine (KR-8900; Topcon, Tokyo, Japan), measurement of IOP (TX-F; Topcon, Tokyo, Japan), slit-lamp bio-microscopy, and color fundus examination. Central corneal thickness, lens thickness, anterior chamber depth, and AL were measured using optical low-coherence reflectometry (Aladdin; Topcon, Japan). Subjective refraction was performed by a trained optometrist for all of the participants. The BCVA was converted into the logarithm of minimal angle resolution (logMAR). Additionally, systolic blood pressure (SBP), diastolic blood pressure (DBP), heart rate, height, and weight were measured. A detailed medical history was also recorded for each participant.

### Swept-Source Optical Coherence Tomography Imaging

The ganglion cell complex (GCC) thicknesses and retinal thicknesses (RT) were measured using SS-OCT (DRI OCT-1 Atlantis; Topcon, Tokyo, Japan), which had a lateral resolution of 10 μm and an axial resolution of 8 μm. The tomography thickness map of the entire macular area (6 × 6 mm) was obtained from an average of four overlapped consecutive scans. The segmentation of each layer was automatically carried out using the built-in software, and manual segmentation was performed where the automatic segmentation misjudged the borderline of each layer. All measurements corrected for the magnification effects of refractive error and AL were conducted by a single technician who was expert in taking OCT images. Follow-up mode was performed to ensure the same location for follow-up scans as the baseline measurement. Images with signal strength index ≤ 60 were excluded from the statistical analysis.

### OCTA Imaging

Spectral-domain OCTA imaging was performed using of a Cirrus AngioPlex device (Carl Zeiss Meditec, Inc., Dublin, CA, USA). This instrument had a center wavelength of 840 nm, a bandwidth of 90 nm, an A-scan depth of 2.0 mm in tissue (1,024 pixels), a full width at half maximum (FWHM) axial resolution of ~5 lm in tissue, a lateral resolution at the retinal surface estimated at ~15 lm, and a scanning rate of 68,000 A-scans per second. All scans were centered on the fovea at two visits using follow-up model after correcting for the magnification effects of refractive error and AL, and FastTrac motion correction software (Carl Zeiss Meditec, Inc., Dublin, CA, USA) was used while the images were acquired (16, 17). The OCTA scans with a signal strength index <40 and images with segmentation errors or residual motion artifacts were excluded.

**Figure 1:**
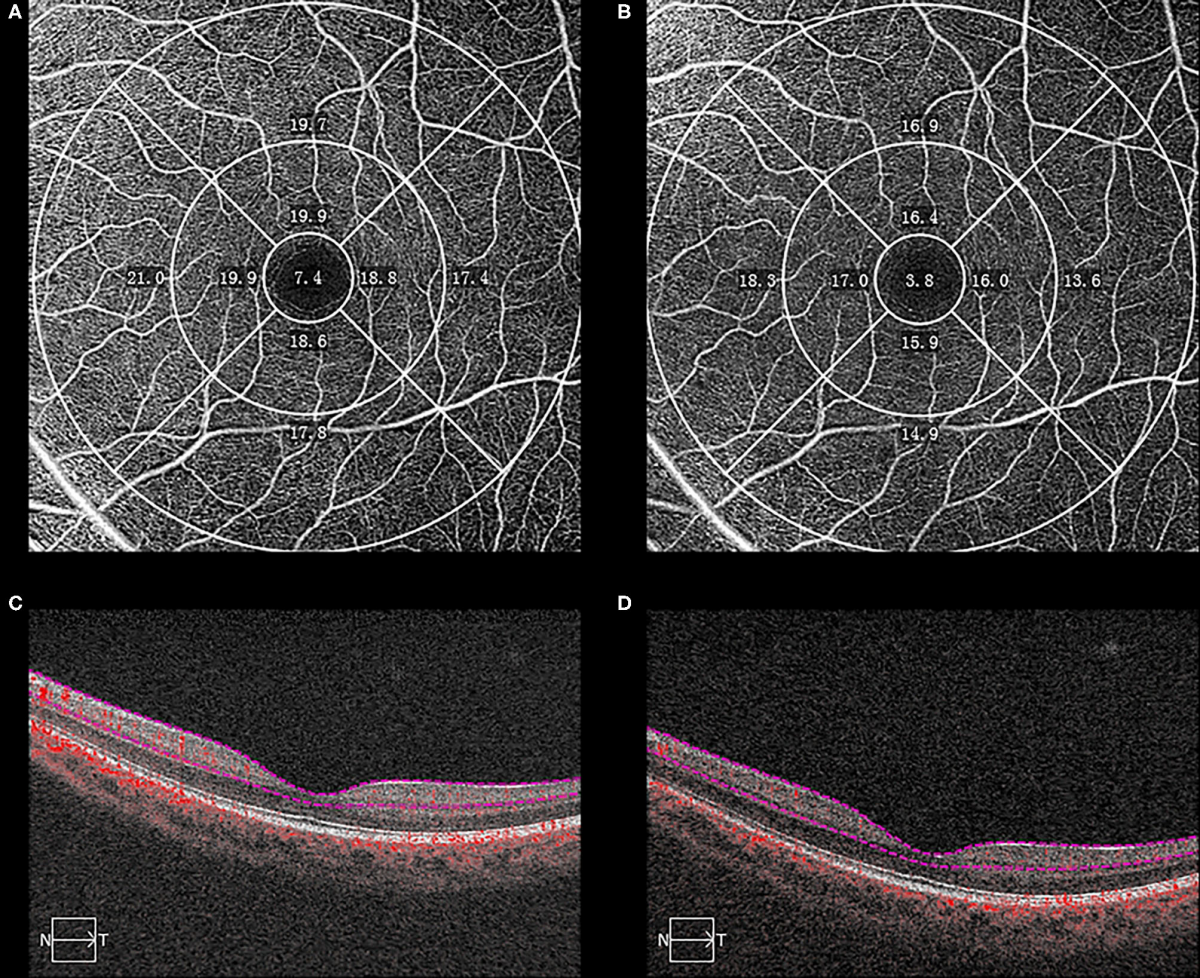
Optical coherence tomography angiographic (OCTA) images from a 18-year-old student with myopia who was followed-up from October 2016 to July 2018. The macular vessel density (VD) decreased between baseline **(A)** and the end of follow-up **(B)** in each sector. The boundaries used for segmentation were indicated between two red lines on the cross-sectional OCTA reflectance **(C,D)**. The sectors were automatically measured according to the Early Treatment of Diabetic Retinopathy Study grid.

Quantitative analysis was undertaken using Cirrus HD-OCT Review Software (version 10.0.0.14618) according to the Early Treatment Diabetic Retinopathy Study (ETDRS) grid (Figure 1). The diameters of the central foveal circle, inner ring, and outer ring were 1, 3, and 6 mm, respectively, and they were further divided into nine sectors (center, IS-inner superior, IN-inner nasal, II-inner inferior, IT-inner temporal, OS-outer superior, ON-outer nasal, OI-outer inferior, OT-outer temporal). The vessel density (VD) was defined as the total length of perfused vasculature per unit area in a region of measurement. The inner plexiform layer boundary was calculated as 70% of the distance from the internal limiting membrane to an estimated boundary of the outer plexiform layer, which was automatically detected by the software.

### Statistical Analysis

In the present study, only the right eye of each participant was selected for statistical analysis. SE was calculated as the sphere plus half a cylinder. The mean arterial pressure (MAP) was calculated according to the following equation: MAP = DBP + 1/3 (SBP-DBP) (18). Mean ocular perfusion pressure (MOPP) was calculated as follows: MOPP = (2/3 × MAP – IOP) (19). The body mass index (BMI) was formulated in the following: weight (kg)/[height (m)]^2^. All participants were divided into three groups by the length of ocular axis at baseline as follows: emmetropia (EM) group with an AL of 24 mm or less; mild-to-moderate myopia (MIM/MOM) group with an AL between 24 and 26 mm; and HM group with an AL of 26 mm or more.

Demographic and ocular parameters were reported as counts or proportions for categorical data, and as mean ± standard deviation for continuous data. The normal distribution of all variables was examined using the Kolmogorov-Smirnov test. Cochran-Mantel-Haenszel test, or one-way analysis of variance (ANOVA) with *post-hoc* test (Bonferroni) was performed to detect differences in demographic and ocular parameters at baseline among the three groups, as appropriate. Biometric changes between 2016 (baseline) and 2018 (follow-up) measured using OCTA and SS-OCT were compared using paired *t*-test. The differences in biometric changes across different refraction groups were analyzed using one-way ANOVA test with *post-hoc* test (Bonferroni). The univariate regression analysis was used to investigate the association between changes in VD and ocular parameters. All the variables with a *p*-values <0.05 in the univariate analysis were considered for the multivariable models. After excluding variables that showed multicollinearity, multivariable regression models were constructed to explore the independent factors for the changes in VD. All statistical analyses were performed using SPSS 25.0 software (IBM, Armonk, NY, USA). A *p*-value <0.05 was considered statistically significant.

## Results

Seven hundred sixty participants were recruited at baseline visit, and 447 (58.82%) participants from the same cohort were followed up 21 months later. The loss to follow-up was chiefly due to school graduation. Moreover, 138 participants were further excluded at follow-up because of lacking OCTA examination (*n* = 128), poor-quality OCTA images (*n* = 5), and refractive surgery history (*n* = 5) and eventually, a total of 309 participants were involved in the final analysis. In the study, 40 participants had EM (12.95%), 194 had MIM or MOM (62.78%), and 75 had HM (24.27%). Demographic and ocular characteristics of participants at baseline are expressed in Table 1. There were no significant differences in age, BMI, SBP, DBP, heart rate, IOP, MOPP, central corneal thickness, and lens thickness among the three groups. However, HM group included fewer female participants (*p* < 0.05), presented significantly lower SE, worse BCVA, deeper ACD, and longer AL than EM group (all *p* < 0.05).

**Table 1:** Demographic and ocular characteristics of study participants.

**Variables**	**EM (*N* = 40)**	**MIM/MOM (*N* = 194)**	**HM (*N* = 75)**	***p*-value**	***Post-hoc***
Age, years	19.73 ± 2.18	19.34 ± 2.27	19.25 ± 2.16	0.2336	/
Female, *n* (%)	27 (67.50)	100 (51.55)	27 (36.00)	0.0042	EM > HM
BMI, kg/m^2^	20.73 ± 3.08	21.02 ± 2.73	20.77 ± 3.43	0.2375	/
SBP, mmHg	120.73 ± 17.55	120.68 ± 15.53	124.55 ± 16.24	0.2212	/
DBP, mmHg	73.70 ± 10.92	71.28 ± 10.11	73.85 ± 9.68	0.0886	/
HR, bpm	74.77 ± 10.27	74.35 ± 11.23	75.56 ± 9.85	0.6828	/
SE, D	−1.49 ± 1.69	−3.70 ± 1.95	−5.97 ± 2.38	<0.0001	EM > MIM/MOM > HM
BCVA, logMAR	0.00 ± 0.00	0.02 ± 0.06	0.03 ± 0.05	0.0004	EM < MIM/MOM, HM
IOP, mmHg	14.05 ± 3.10	13.98 ± 2.69	14.00 ± 2.80	0.9683	/
MOPP, mmHg	45.77 ± 8.54	44.54 ± 7.61	44.89 ± 12.06	0.2886	/
ACD, mm	3.60 ± 0.21	3.74 ± 0.22	3.77 ± 0.27	0.0004	EM < MIM/MOM, HM
CCT, μm	543.06 ± 35.72	539.82 ± 35.04	535.84 ± 37.34	0.5862	/
LT, mm	3.53 ± 0.22	3.49 ± 0.36	3.49 ± 0.22	0.5699	/

*Data are presented as mean ± standard deviation unless otherwise indicated*.

*Comparison among the three groups were using the Cochran-Mantel-Haenszel test for categorical data or the one-way ANOVA test for continuous data with post-hoc test (Bonferroni)*.

*ACD, anterior chamber depth; BCVA, best-corrected visual acuity; BMI, body mass index; CCT, central corneal thickness; DBP, diastolic blood pressure; EM, emmetropia; HM, high myopia; HR, heart rate; IOP, intraocular pressure; logMAR, logarithm of minimal angle resolution; LT, lens thickness; MIM, mild myopia; MOM, moderate myopia; MOPP, mean ocular perfusion pressure; SBP, systolic blood pressure; SE, spherical equivalent*.

**Figure 2:**
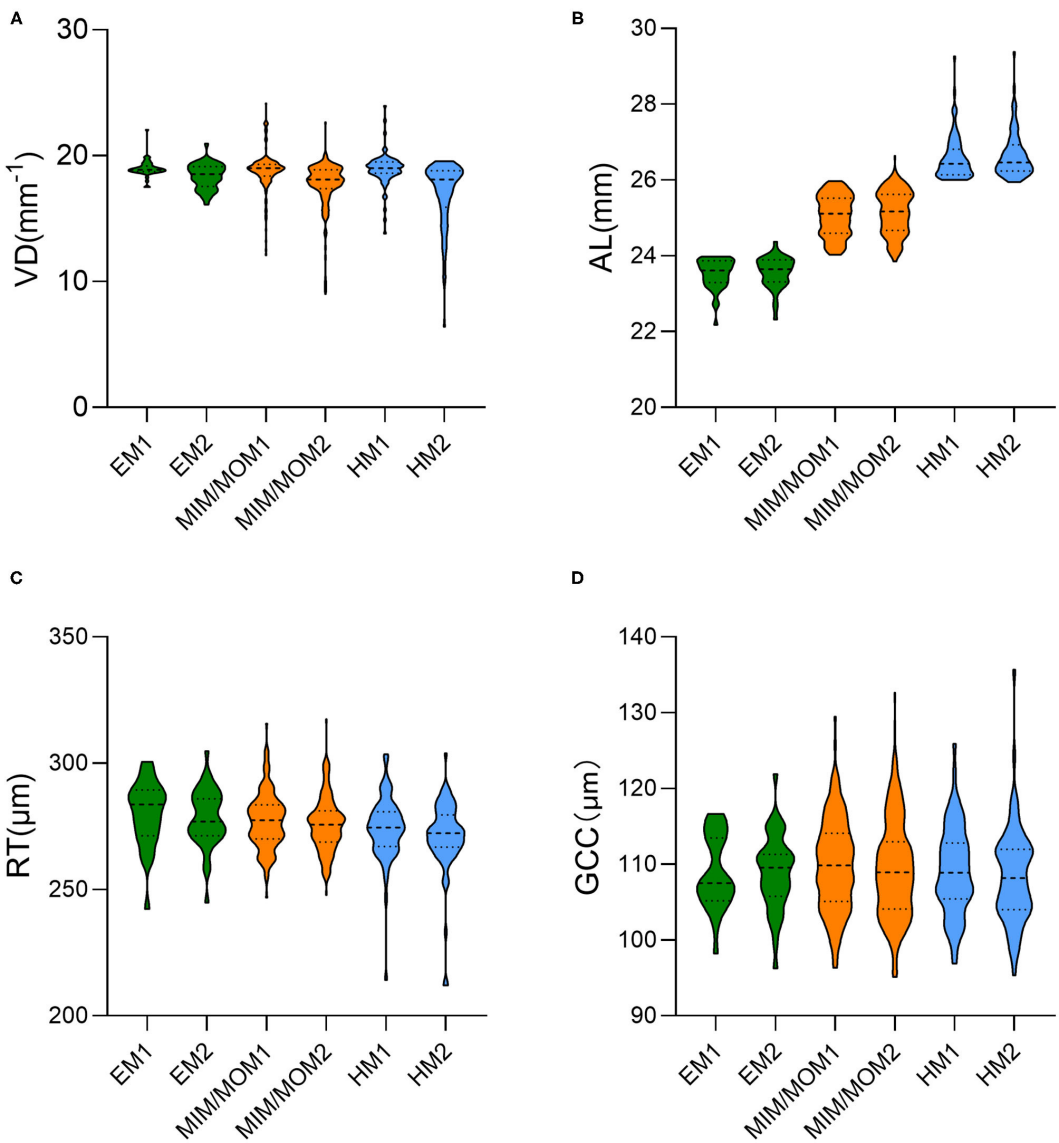
The violin plot in distribution of **(A)** vessel density (VD), **(B)** axial length (AL), **(C)** retinal thickness (RT) and **(D)** ganglion cell complex (GCC) thickness in different groups at baseline and the end of follow-up. EM1, emmetropia at baseline; EM2, emmetropia at the end of follow-up; HM1, high myopia at baseline; HM2, high myopia at the end of follow-up; MIM/MOM1, mild-to-moderate myopia at baseline; MIM/MOM2, mild-to-moderate myopia at the end of follow-up.

Table 2 and Figure 2 demonstrates the baseline and the longitudinal changes of VD and ocular parameters over follow-up period. There was no significant difference in the baseline VD, RT and GCC among the three groups (all *p* > 0.05). The macular VD significantly decreased while AL increased in the follow-up in all the three groups (all *p* < 0.05). Of note, the HM group showed a larger reduction in VD (−1.96 ± 2.57/mm) compared with the EM group (−0.61 ± 0.97/mm) (*p* = 0.0307). There were reductions in both RT and GCC thickness after 2-year follow-up in HM and MIM/MOM groups (both *p* < 0.01), which was not seen in emmetropic eyes (both *p* > 0.05).

**Table 2:** Comparison of globally VD and ocular parameters between baseline and the end of follow-up period.

	**EM (*N* = 40)**	**MIM/MOM (*N* = 194)**	**HM (*N* = 75)**	***p*-value**	***Post-hoc***
VD, mm
Baseline	19.00 ± 0.71	18.82 ± 1.52	18.95 ± 1.36	0.5843	/
Follow-up	18.39 ± 1.01	17.99 ± 4.87	17.01 ± 2.60	0.1392	/
Changes	−0.61 ± 0.97	−0.81 ± 5.08	−1.96 ± 2.57	0.0307	EM < HM
*p*-value	0.0003	<0.0001	<0.0001		
AL, mm
Baseline	23.53 ± 0.40	25.06 ± 0.54	26.59 ± 0.59	<0.0001	EM < MIM/MOM < HM
Follow-up	23.56 ± 0.41	25.15 ± 0.58	26.67 ± 0.62	<0.0001	EM < MIM/MOM < HM
Changes	0.04 ± 0.10	0.08 ± 0.18	0.08 ± 0.13	0.0384	EM < MIM/MOM, HM
*p*-value	0.0253	<0.0001	<0.0001		
RT, μm
Baseline	280.08 ± 12.96	277.33 ± 11.05	273.99 ± 13.13	0.0530	/
Follow-up	278.29 ± 11.46	275.80 ± 10.74	267.13 ± 35.02	0.0052	EM, MIM/MOM > HM
Changes	−0.79 ± 2.97	−1.86 ± 3.32	−6.94 ± 32.17	0.1585	/
*p*-value	0.1783	<0.0001	0.0006		
GCC, μm
Baseline	108.92 ± 4.88	109.86 ± 6.04	109.19 ± 5.89	0.6318	/
Follow-up	108.88 ± 5.00	109.32 ± 6.54	105.25 ± 16.48	0.3053	/
Changes	−0.14 ± 2.19	−0.46 ± 2.99	−3.66 ± 15.34	0.2452	/
*p*-value	0.7382	<0.0001	0.0031		

*Data are presented as mean ± standard deviation*.

*Comparisons between the baseline and the follow-ups using the paired t-test. Comparison among the three groups were using the one-way ANOVA test with post-hoc test (Bonferroni)*.

*EM, emmetropia; GCC, ganglion cell complex; MIM, mild myopia, MOM, moderate myopia; HM, high myopia; RT, retinal thickness; VD, macular vessel density*.

**Figure 3:**
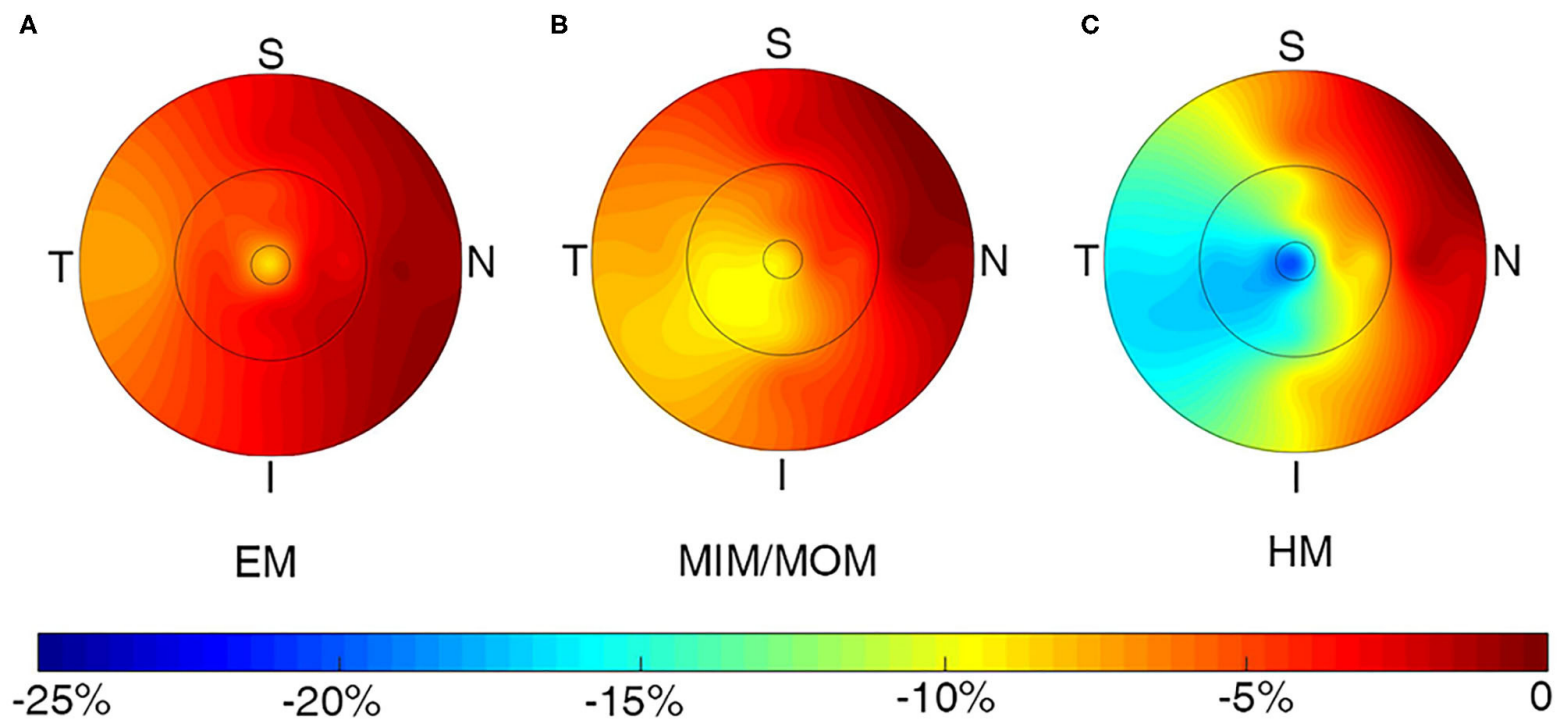
Topographic variation of the change rate of macular vessel density in all sectors for **(A)** EM, **(B)** MIM/MOM, **(C)** HM. EM, emmetropia; HM, high myopia; MIM/MOM, mild-to-moderate myopia.

The changes of macular VD in all sectors of ETDRS grid were presented in Supplementary Table 1 and Figure 3. The sectoral VD significantly decreased through follow-up in all the three groups (all *p* < 0.05), except for that in the outer nasal sector in EM group (*p* = 0.2521). In each group, the nasal sector minimally changed, whereas the temporal sector maximally changed in both inner ring and outer ring. In addition, the changes in the inner ring were more significant than those in the outer ring for the majority of sectors. Compared with EM group, HM group had notably higher change rate in the II sector (−3.15 ± 9.62% vs. −14.88 ± 20.11%, *p* = 0.0073), IT sector (−4.88 ± 8.43% vs. −17.22 ± 22.88%, *p* = 0.0298), and OT sector (−7.15 ± 8.40% vs. −16.20 ± 20.80%, *p* = 0.0308).

**Figure 4:**
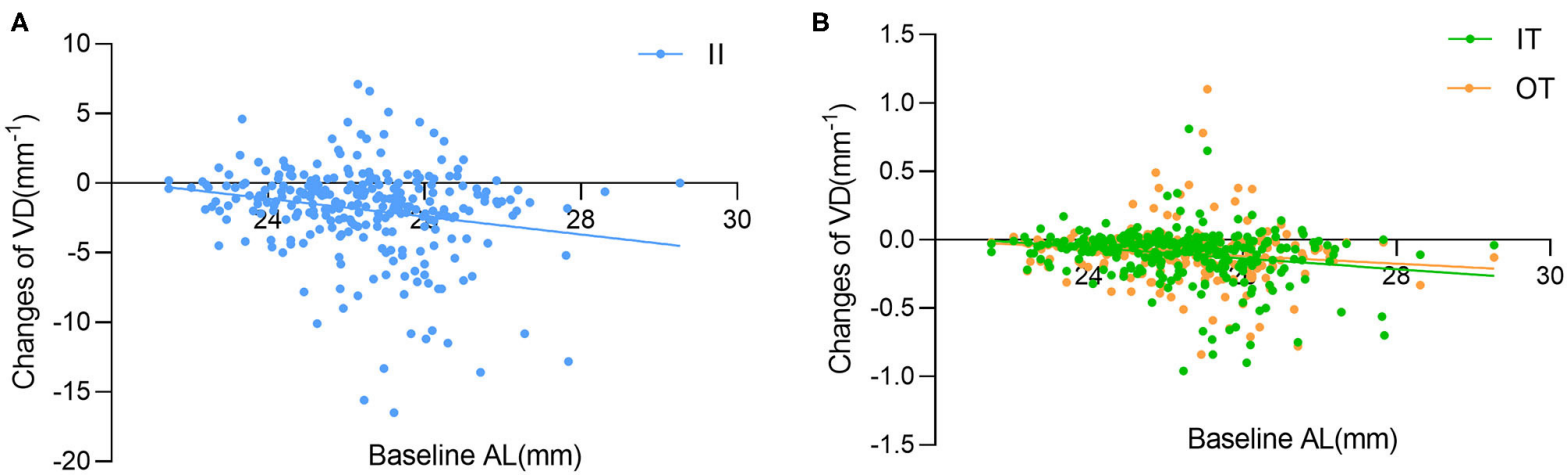
Simple linear regression analysis between baseline AL with the changes of vessel density (VD) in the **(A)** inner inferior (II), **(B)** inner temporal (IT) and outer temporal (OT) sectors.

The results of the univariate regression analysis adjusted for age and gender are presented in Supplementary Tables 2–5. For the average macular VD, no variable was found to be correlated with the changes of VD (all *p* > 0.05). The associations of macular VD changes with other ocular parameters were further explored in selected sectors where great macular VD reduction were observed in HM group. The changes of VD significantly correlated with SE, baseline AL and the changes of AL in the II, IT, and OT sectors. BCVA and the changes of GCC thickness were negatively associated with the changes of VD in the II and IT sectors. Additionally, the changes of VD were positively correlated with baseline RT and the changes of RT in the II sector, whereas negatively correlated with ACD in the OT sector. Variables with a significance *p*-value <0.05 were included in the stepwise multivariate models except for SE that had a stronger correlation with AL. The results of multivariable regression analysis (Table 3 and Figure 4) revealed the baseline AL was independently associated with the changes of VD in II, IT, and OT sectors (all *p* < 0.05). According to the models, each 1 mm increase in baseline AL was associated with a 0.57/mm decrease in inner-inferior macular VD, a 0.73/mm decrease in inner-temporal macular VD, and a 0.44/mm decrease in outer-temporal macular VD, separately.

**Table 3:** Multivariate regression analysis of association with changes of sectoral VD in all participants.

**Model**	**Coefficient estimate**	**95% confidence interval**	***p*-value**
II
BCVA, logMAR	3.37	−2.31 to 9.05	0.2430
Baseline AL, mm	−0.57	−1.05 to −0.08	0.0224
Changes of AL, mm	−2.59	−5.67 to 0.50	0.0995
Baseline RT, μm	0.02	−0.03 to 0.06	0.4354
Changes of RT, μm	0.02	−0.03 to 0.08	0.3874
Changes of GCC, μm	0.02	−0.10 to 0.14	0.7346
IT
BVCA, logMAR	1.45	−4.62 to 7.51	0.6379
Baseline AL, mm	−0.73	−1.25 to −0.21	0.0057
Changes of AL, mm	−2.46	−5.76 to 0.85	0.1445
Changes of GCC, μm	0.05	−0.01 to 0.11	0.1281
OT
ACD, mm	−1.31	−2.92 to 0.30	0.1095
Baseline AL, mm	−0.44	−0.82 to −0.05	0.0266
Changes of AL, mm	−2.10	−4.62 to 0.42	0.1022

*ACD, anterior chamber depth; AL, axial length; BCVA, best-corrected visual acuity; GCC, ganglion cell complex; II, inner inferior; IT, inner temporal; logMAR, logarithm of minimal angle resolution; OT, outer temporal; RT, retinal thickness; VD, vessel density*.

## Discussion

This prospective study, which enrolled three hundred and nine university students over 21 months, was the first longitudinal study evaluating macular microvasculature changes in young adults observed by OCTA. A significant decrease in macular VD over time was documented. In high myopes, macular VD presented larger reduction globally and in the II, IT and OT sectors. A longer baseline AL was associated with greater loss of VD in these sectors.

Earlier studies reported that age was determinant of macular VD measured by OCTA (20–22). However, these studies were generally conducted in healthy eyes. In our longitudinal study, macular microvasculature was significantly decreased in both emmetropes and myopes over a 21-month period. Jo et al. (20) reported that the majority of the sectoral peripapillary and macular VDs were significantly reduced with increase of age in healthy eyes but the VD of papillomacular bundle area was not age-dependent. The present study also demonstrated that the macular VD in the ON sector did not decrease in emmetropic students, while this sectoral VD reduced in myopic students, which indicating the effect of myopia on macular perfusion reflected in the area of papillomaular bundle area. It was revealed that the ischemic injury of the papillomacular bundle could predict poor vision in both central retinal artery occlusion and branch retinal artery occlusion (23, 24). Thus, the effects of VD loss in the papillomacular bundle area on visual impairment in myopes and its underlying mechanism still require further investigations.

Several studies used OCTA to investigate the changes of retinal microvascular in HM, and they have reported controversial findings (13, 25–28). Guo et al. (28) pointed out that HM causes a lower superficial peripapillary microvascular density, but no significant difference was found in parafoveal microvascular density among all groups. Wang et al. (26) also found a decreased vessel density in the peripapillary area, rather than in the parafoveal area of high myopic eyes. On the contrary, our previous study and a number of cross-sectional studies (13, 25, 27) have yielded a reduced parafoveal microvascular density in HM. It may lead to controversial results because the vessel density was quantified in different ways. In Guo's and Wang's studies, the vessel density was defined as the proportion of the total area occupied by vessels, while the vessel density was defined as the total length of perfused vasculature per unit area in this study. Moreover, the previous studies were all cross-sectional studies with different simple sizes. The present research prospectively evaluated the changes of macular VD and showed a significantly greater loss of VD in HM group than in EM group. However, no significant difference of global and sectoral VD changes was noted between MIM/MOM group and HM group, which was in line with Yang et al.'s findings (29). When it comes to sectoral changes of macular VD, there was no unified understanding. In the comparison with EM group, HM group presented larger longitudinal changes of macular VD in the II, IT, and OT sectors in this study, indicating these sectors might be critical focus areas in clinical practice.

We previously compared the peripapillary and parafoveal vessel density with AL and retina thickness, and found a significant negative correlation between AL and vessel density (13). Li et al. (27) pointed out that microvascular density was negatively correlated with AL in myopia in both superficial and deep vascular plexuses. Ucak et al. (30) reported that the macular vessel density reduced with increase of AL and decrease of GCC thicknesses in patients with HM. The current study uncovered that the longitudinal changes of macular VD were only correlated with baseline AL in the II, IT, and OT sectors, suggesting the macular vessel loss mainly results from the elongation of eyeballs. It is suggested that with the increase of AL in myopia, the eyeball stretches, causing mechanically expanding and thinning of retina, resulting in a narrowing of the vessel diameter, which leads to the decrease of vessel density (27). Meanwhile, the macular thickness showed different rates of change in different sectors according to age and refractive status (31, 32). Since the oxygen demands of those sectors might reduce resulting from the thinning of retina, the vessels supplying blood to those regions was likely to decrease accordingly. As well as the mechanical stretch, there might be some changes in myopia-related signaling pathways, resulting in subsequent changes in vessel density. However, it is not clear which of structural changes or biochemical changes came first. Despite this, the mechanisms of sectoral changes in macular vessel density still require further study.

The capillary networks in the human retina mainly distributed in the inner five layers of the retina, of which the superficial capillary network is mainly distributed in the nerve fiber layer and the ganglion cell layer, while the deep capillaries are mainly distributed in the inner plexiform layer and the inner nuclear layer (33). There has been increasing evidence that HM has longer AL, lower peripapillary VD, and thinner peripapillary retinal nerve fiber layer thickness (26, 28, 34, 35). In addition, AL was also noted to be negatively correlated with thickness of the outer nuclear layer and photoreceptor outer segment layer (36). But the RT and GCC thickness were not related with the loss of macular VD in our multivariate regression analysis. The underlying biochemical mechanism might give reason to changes of macular VD as well as the mechanical stretch. Dopamine, secreted by dopaminergic amacrine cell (DAC) in the inner layer of the retina, was indicated to be a key molecule in the retinal signaling pathway during myopia development, working with Melatonin and retinal ganglion cells (RGCs) (37–39). The structural changes in myopia may affect the DAC, RGCs, and outer photoreceptor cells, leading to subsequent changes in DA and melatonin synthesis and release, thereby influencing further development of myopia.

This study has several limitations. Firstly, the low follow-up rate made it not representative. Secondly, the examinations were only performed at two visits, so that we could not observe the dynamic changes during this period. Thirdly, the small sample size, especially for EM and HM groups, may significantly impact the reliability of our findings. Fourthly, OCTA deep slab images were not included in the analysis because several projection artifacts prevented qualified images.

In conclusion, the macular VD significantly decreased over time in all the sectors in young myopes, while the macular VD in the papillomacular bundle area was not age-dependent in emmetropes. A larger reduction of macular VD was observed in HM group compared with healthy eyes and correlated with longer baseline AL in the II, IT and OT sectors. Although the underlying mechanisms of physiological and pathological macular VD changes remain unknown, the reduction of macular VD might be considered as a preclinical characteristic in patients with HM. Therefore, further studies with longer follow-up periods are needed to investigate correlations between structure and function.

## Data Availability Statement

The raw data supporting the conclusions of this article will be made available by the authors, without undue reservation.

## Ethics Statement

The studies involving human participants were reviewed and approved by Ethical committee of Shanghai General Hospital, Shanghai Jiao Tong University, Shanghai, China. The patients/participants provided their written informed consent to participate in this study.

## Author Contributions

YF, JZ, JH, and XX: study concept and design. YS, QC, GH, and YY: data collection and management. YS and LY: analysis and interpretation of data. YS: writing the manuscript. JZ, JH, ZZ, and HZ: critical revision of the manuscript. XX: supervision. All authors read and approved the final manuscript.

## Conflict of Interest

The authors declare that the research was conducted in the absence of any commercial or financial relationships that could be construed as a potential conflict of interest.

## Abbreviations

ACD: anterior chamber depth
AL: axial length
BCVA: best-corrected visual acuity
BMI: body mass index
CCT: central corneal thickness
DBP: diastolic blood pressure
EM: emmetropia
ETDRS: Early Treatment Diabetic Retinopathy Study
GCC: ganglion cell complex
HM: high myopia
HR: heart rate
II: inner inferior
IN: inner nasal
IOP: intraocular pressure
IS: inner superior
IT: inner temporal
logMAR: logarithm of the minimum angle of resolution
LT: lens thickness
MIM: mild myopia
MOM: moderate myopia
MOPP: mean ocular perfusion pressure
OI: outer inferior
ON: outer nasal
OS: outer superior
OT: outer temporal
RT: retinal thickness
SBP: systolic blood pressure
SD: standard deviation
SE: spherical equivalent
VD: vessel density.
