# Supplementary material for: Macular Vessel Density Changes in Young Adults With High Myopia: A Longitudinal Study
Source: Front Med (Lausanne). 2021 Jun 8;8:648644. doi: 10.3389/fmed.2021.648644 (PMC8217628; doi:10.3389/fmed.2021.648644)
Supplement: Supplementary file 1 [file Table_1.DOCX]

**Supplementary Table S1** Comparison of sectoral vessel density between baseline and the end of follow-up period.

| Sectors | EM (N=40) | MIM/MOM (N=194) | HM (N=75) | *p* value | Post Hoc |
| --- | --- | --- | --- | --- | --- |
| Center |  |  |  |  |  |
| Baseline | 9.59±2.26 | 10.23±3.29 | 10.94±3.02 | 0.0350 | EM < HM |
| Follow-up | 8.86±3.45 | 8.75±3.61 | 8.68±4.10 | 0.9589 | / |
| Change rate, % | -8.80±24.33 | -8.97±46.73 | -20.00±35.64 | 0.2871 | / |
| *p* value | 0.0463 | <0.0001 | <0.0001 |  |  |
| IS |  |  |  |  |  |
| Baseline | 19.31±0.99 | 19.24±1.84 | 19.30±1.79 | 0.7486 | / |
| Follow-up | 18.35±1.77 | 17.94±2.55 | 17.20±3.03 | 0.2331 | / |
| Change rate, % | -4.86±9.00 | -5.79±16.38 | -10.32±17.09 | 0.4764 | / |
| *p* value | 0.0026 | <0.0001 | <0.0001 |  |  |
| IN |  |  |  |  |  |
| Baseline | 19.00±0.96 | 19.05±2.11 | 19.06±1.95 | 0.5391 | / |
| Follow-up | 18.44±1.52 | 17.91±2.85 | 17.23±3.45 | 0.3552 | / |
| Change rate, % | -2.80±8.23 | -4.63±20.12 | -8.78±20.03 | 0.8728 | / |
| *p* value | 0.0402 | <0.0001 | 0.0039 |  |  |
| II |  |  |  |  |  |
| Baseline | 18.94±1.41 | 18.81±1.94 | 18.95±1.63 | 0.8837 | / |
| Follow-up | 18.26±1.37 | 17.01±2.95 | 16.05±3.59 | 0.0026 | EM > HM |
| Change rate, % | -3.15±9.62 | -8.74±17.93 | -14.88±20.11 | 0.0073 | EM > HM |
| *p* value | 0.0239 | <0.0001 | <0.0001 |  |  |
| IT |  |  |  |  |  |
| Baseline | 19.11±1.26 | 18.83±1.99 | 18.88±1.79 | 0.3831 | / |
| Follow-up | 18.14±1.56 | 16.93±3.06 | 15.53±4.16 | 0.0017 | EM > HM |
| Change rate, % | -4.88±8.43 | -9.09±19.48 | -17.22±22.88 | 0.0298 | EM > HM |
| *p* value | 0.0010 | <0.0001 | <0.0001 |  |  |
| OS |  |  |  |  |  |
| Baseline | 19.40±0.64 | 19.06±1.30 | 19.12±1.20 | 0.4890 | / |
| Follow-up | 18.89±0.93 | 18.41±1.72 | 17.95±2.48 | 0.2456 | / |
| Change rate, % | -2.57±5.02 | -2.79±13.87 | -5.86±11.45 | 0.5976 | / |
| *p* value | 0.0047 | <0.0001 | <0.0001 |  |  |
| ON |  |  |  |  |  |
| Baseline | 20.35±0.42 | 20.39±1.07 | 20.55±0.65 | 0.0406 | EM < HM |
| Follow-up | 20.16±0.64 | 20.11±1.88 | 20.08±1.49 | 0.1678 | / |
| Change rate, % | -0.92±3.18 | -0.90±10.10 | -1.99±6.26 | 0.8072 | / |
| *p* value | 0.2521 | 0.0270 | 0.0432 |  |  |
| OI |  |  |  |  |  |
| Baseline | 18.98±1.10 | 18.80±1.51 | 18.92±1.78 | 0.5202 | / |
| Follow-up | 18.35±1.19 | 17.59±2.23 | 16.98±2.70 | 0.0369 | EM > HM |
| Change rate, % | -2.94±9.16 | -5.64±14.31 | -9.52±13.29 | 0.0837 | / |
| *p* value | 0.0051 | <0.0001 | <0.0001 |  |  |
| OT |  |  |  |  |  |
| Baseline | 18.42±0.81 | 17.47±2.21 | 17.40±2.15 | 0.0190 | EM > MIM/MOM/HM |
| Follow-up | 17.10±1.72 | 15.90±2.74 | 14.49±3.70 | 0.0003 | EM > MIM/MOM > HM |
| Change rate, % | -7.15±8.40 | -7.62±20.80 | -16.20±20.80 | 0.0308 | EM > HM |
| *p* value | <0.0001 | <0.0001 | <0.0001 |  |  |
| Data are presented as mean± standard deviation.  Comparison among the three groups were using the Cochran-Mantel-Haenszel test for categorical data or the one-way ANOVA test for continuous data with post hoc test (Bonferroni).  *EM emmetropia, II inner inferior, IN inner nasal, IS inner superior, IT inner temporal, MIM mild myopia, MOM moderate myopia, OI outer inferior, ON outer nasal, OS outer superior, OT outer temporal.* | | | | | |

**Supplementary Table S2** Results of univariable regression analysis – dependent variable: changes of global VD (/mm).

| Model | Coefficient estimate | 95% confidence interval | *p* value |
| --- | --- | --- | --- |
| BMI, kg/m^2^ | -0.04 | -0.21 to 0.13 | 0.6503 |
| SBP, mmHg | -0.003 | -0.040 to 0.033 | 0.8602 |
| DBP, mmHg | 0.02 | -0.03 to 0.08 | 0.3663 |
| HR, bpm | 0.02 | -0.02 to 0.07 | 0.3268 |
| SE, D | 0.16 | -0.04 to 0.36 | 0.1125 |
| BCVA, logMAR | -4.36 | -13.80 to 5.08 | 0.3638 |
| IOP, mmHg | 0.06 | -0.11 to 0.24 | 0.4773 |
| MOPP, mmHg | 0.002 | -0.055 to 0.059 | 0.9430 |
| ACD, mm | -1.79 | -3.99 to 0.40 | 0.1091 |
| CCT, µm | 0.01 | -0.003 to 0.026 | 0.1264 |
| LT, mm | 0.009 | -1.532 to 1.794 | 0.8769 |
| Baseline AL, mm | -0.35 | -0.81 to 0.12 | 0.1409 |
| Changes of AL, mm | -2.83 | -6.05 to 0.39 | 0.0845 |
| Baseline RT, µm | -0.01 | -0.06 to 0.04 | 0.7058 |
| Changes of RT, µm | 0.02 | -0.02 to 0.05 | 0.3750 |
| Baseline GCC, µm | 0.02 | -0.09 to 0.12 | 0.7592 |
| Changes of GCC, µm | 0.04 | -0.04 to 0.11 | 0.3128 |
| *ACD anterior chamber depth, AL axial length, BCVA best-corrected visual acuity, BMI body mass index, CCT central corneal thickness, DBP diastolic blood pressure, GCC ganglion cell complex, HR heart rate, IOP intraocular pressure, logMAR logarithm of minimal angle resolution, LT lens thickness, MOPP mean ocular perfusion pressure, RT retinal thickness, SBP systolic blood pressure, SE spherical equivalent, VD vessel density.* | | | |

**Supplementary Table S3** Results of univariable regression analysis – dependent variable: changes of inner inferior VD (/mm)

| Model | Coefficient estimate | 95% confidence interval | *p* value |
| --- | --- | --- | --- |
| BMI, kg/m^2^ | 0.07 | -0.07 to 0.21 | 0.3103 |
| SBP, mmHg | -0.006 | -0.036 to 0.024 | 0.7079 |
| DBP, mmHg | -0.005 | -0.047 to 0.036 | 0.7964 |
| HR, bpm | -0.02 | -0.06 to 0.02 | 0.3765 |
| SE, D | 0.24 | 0.08 to 0.40 | 0.0032 |
| BCVA, logMAR | -8.70 | -15.92 to -1.47 | 0.0185 |
| IOP, mmHg | 0.07 | -0.07 to 0.22 | 0.3094 |
| MOPP, mmHg | -0.02 | -0.07 to 0.03 | 0.4330 |
| ACD, mm | -0.94 | -2.69 to 0.81 | 0.2911 |
| CCT, µm | 0.007 | -0.005 to 0.019 | 0.2278 |
| LT, mm | 0.35 | -0.94 to 1.63 | 0.5941 |
| Baseline AL, mm | -0.64 | -1.02 to -0.26 | 0.0009 |
| Changes of AL, mm | -2.91 | -5.50 to -0.31 | 0.0282 |
| Baseline RT, µm | 0.04 | 0.002 to 0.078 | 0.0416 |
| Changes of RT, µm | 0.04 | 0.01 to 0.07 | 0.0049 |
| Baseline GCC, µm | 0.02 | -0.06 to 0.09 | 0.6954 |
| Changes of GCC, µm | 0.08 | 0.03 to 0.14 | 0.0040 |
| *ACD anterior chamber depth, AL axial length, BCVA best-corrected visual acuity, BMI body mass index, CCT central corneal thickness, DBP diastolic blood pressure, GCC ganglion cell complex, HR heart rate, IOP intraocular pressure, logMAR logarithm of minimal angle resolution, LT lens thickness, MOPP mean ocular perfusion pressure, RT retinal thickness, SBP systolic blood pressure, SE spherical equivalent, VD vessel density.* | | | |

**Supplementary Table S4** Results of univariable regression analysis – dependent variable: changes of inner temporal VD (/mm)

| Model | Coefficient estimate | 95% confidence interval | *p* value |
| --- | --- | --- | --- |
| BMI, kg/m^2^ | 0.15 | -0.001 to 0.301 | 0.0513 |
| SBP, mmHg | 0.008 | -0.025 to 0.041 | 0.6181 |
| DBP, mmHg | 0.002 | -0.043 to 0.047 | 0.9351 |
| HR, bpm | -0.007 | -0.048 to 0.035 | 0.7441 |
| SE, D | 0.25 | 0.08 to 0.43 | 0.0048 |
| BCVA, logMAR | -11.45 | -19.25 to -3.66 | 0.0041 |
| IOP, mmHg | 0.13 | -0.02 to 0.29 | 0.0909 |
| MOPP, mmHg | -0.01 | -0.07 to 0.04 | 0.6296 |
| ACD, mm | -0.90 | -2.79 to 1.00 | 0.3523 |
| CCT, µm | 0.01 | -0.003 to 0.022 | 0.1290 |
| LT, mm | 0.03 | -1.37 to 1.42 | 0.9696 |
| Baseline AL, mm | -0.73 | -1.14 to -0.32 | 0.0006 |
| Changes of AL, mm | -2.94 | -5.78 to -0.11 | 0.0421 |
| Baseline RT, µm | 0.03 | -0.007 to 0.074 | 0.1043 |
| Changes of RT, µm | 0.03 | -0.001 to 0.056 | 0.0630 |
| Baseline GCC, µm | 0.02 | -0.07 to 0.10 | 0.7229 |
| Changes of GCC, µm | 0.06 | 0.001 to 0.118 | 0.0465 |

*ACD anterior chamber depth, AL axial length, BCVA best-corrected visual acuity, BMI body mass index, CCT central corneal thickness, DBP diastolic blood pressure, GCC ganglion cell complex, HR heart rate, IOP intraocular pressure, logMAR logarithm of minimal angle resolution, LT lens thickness, MOPP mean ocular perfusion pressure, RT retinal thickness, SBP systolic blood pressure, SE spherical equivalent, VD vessel density.*

**Supplementary Table S5** Results of univariable regression analysis – dependent variable: changes of outer temporal VD (/mm)

| Model | Coefficient estimate | 95% confidence interval | *p* value |
| --- | --- | --- | --- |
| BMI, kg/m^2^ | 0.09 | -0.04 to 0.22 | 0.1584 |
| SBP, mmHg | 0.02 | -0.004 to 0.052 | 0.0976 |
| DBP, mmHg | 0.04 | -0.006 to 0.077 | 0.0957 |
| HR, bpm | 0.005 | -0.031 to 0.041 | 0.7852 |
| SE, D | 0.17 | 0.01 to 0.33 | 0.0368 |
| BCVA, logMAR | -4.51 | -13.31 to 4.30 | 0.3138 |
| IOP, mmHg | 0.01 | -0.12 to 0.15 | 0.8375 |
| MOPP, mmHg | 0.04 | -0.008 to 0.085 | 0.1033 |
| ACD, mm | -1.76 | -3.37 to -0.16 | 0.0311 |
| CCT, µm | 0.003 | -0.008 to 0.014 | 0.5576 |
| LT, mm | 0.99 | -0.63 to 2.60 | 0.2296 |
| Baseline AL, mm | -0.46 | -0.82 to -0.09 | 0.0144 |
| Changes of AL, mm | -2.61 | -5.08 to -0.14 | 0.0385 |
| Baseline RT, µm | 0.003 | -0.032 to 0.038 | 0.8607 |
| Changes of RT, µm | 0.01 | -0.01 to 0.04 | 0.3106 |
| Baseline GCC, µm | -0.01 | -0.08 to 0.06 | 0.7217 |
| Changes of GCC, µm | 0.02 | -0.03 to 0.07 | 0.3433 |
| *ACD anterior chamber depth, AL axial length, BCVA best-corrected visual acuity, BMI body mass index, CCT central corneal thickness, DBP diastolic blood pressure, GCC ganglion cell complex, HR heart rate, IOP intraocular pressure, logMAR logarithm of minimal angle resolution, LT lens thickness, MOPP mean ocular perfusion pressure, RT retinal thickness, SBP systolic blood pressure, SE spherical equivalent, VD vessel density* | | | |
